# Self-interested learning is more important than fair-minded conditional cooperation in public-goods games

**DOI:** 10.1017/ehs.2022.45

**Published:** 2022-10-17

**Authors:** Maxwell N. Burton-Chellew, Claire Guérin

**Affiliations:** 1 Department of Economics, HEC-University of Lausanne, 1015 Lausanne, Switzerland; 2 Department of Ecology and Evolution, Biophore, University of Lausanne, 1015 Lausanne, Switzerland.

**Keywords:** altruism, behavioural economics, confusion, reciprocity, social preferences

## Abstract

Why does human cooperation often unravel in economic experiments despite a promising start? Previous studies have interpreted the decline as the reaction of disappointed altruists retaliating in response to non-altruists (Conditional Cooperators hypothesis). This interpretation has been considered evidence of a uniquely human form of cooperation, motivated by an altruistic concern for equality (‘fairness’) and requiring special evolutionary explanations. However, experiments have typically shown individuals not only information about the decisions of their groupmates (social information) but also information about their own payoffs. Showing both confounds explanations based on conditional cooperation with explanations based on confused individuals learning how to better play the game (Confused Learners hypothesis). Here we experimentally decouple these two forms of information, and thus these two hypotheses, in a repeated public-goods game. Analysing 616 Swiss university participants, we find that payoff information leads to a greater decline, supporting the Confused Learners hypothesis. In contrast, social information has a small or negligible effect, contradicting the Conditional Cooperators hypothesis. We also find widespread evidence of both confusion and selfish motives, suggesting that human cooperation is maybe not so unique after all.

## Graphical abstract

**Figure:**
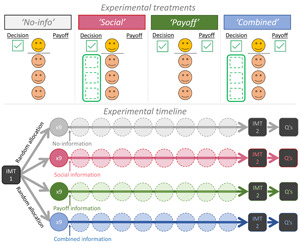

**Social media summary:** Do people strive for equality or personal success? Decoupling conditional cooperation and learning in public-goods games

## Introduction

Sound knowledge about the specific motives behind altruistic acts predominantly stems from laboratory experiments. (Fehr & Fischbacher, 2003)

We need to ask what the totality of [experimental] evidence tells us about the maintained assumptions of theory and its test framework. (Smith, 2010)

Economic experiments using the public-goods game have shown that many people initially make costly contributions towards a group beneficial account (cooperate) but that this cooperation is fragile and typically declines over time (Burton-Chellew & West, 2021; Chaudhuri, 2011; Jouxtel, 2019; Ledyard, 1995; Neugebauer, Perote, Schmidt, & Loos, 2009; Thielmann, Böhm, Ott, & Hilbig, 2021; Zelmer, 2003). There are two major competing explanations for this decline in contributions that are both based on different forms of learning (Andreoni, 1995; Burton-Chellew & West, 2021; Cooper & Stockman, 2002; Houser & Kurzban, 2002). One hypothesis is that cooperation unravels owing to disappointment among cooperative individuals as they learn that people are not as cooperative as they anticipated (Fischbacher & Gachter, 2010; Fischbacher, Gachter, & Fehr, 2001). This ‘Conditional Cooperators’ hypothesis posits that people are mostly motivated by an altruistic concern for equality (or ‘fairness’), and that human cooperation requires a unique evolutionary explanation (Fehr & Fischbacher, 2003; Fehr & Schurtenberger, 2018; Henrich & Muthukrishna, 2021). The competing hypothesis is that cooperation decreases as confused individuals learn that contributing is not as profitable as they thought. This ‘Confused Learners’ hypothesis posits that people are mostly motivated by self-interest but are not well adapted to laboratory games and thus need experience to recalibrate their behaviour (Barclay, 2012; Barclay & Daly, 2003; Bateson, Nettle, & Roberts, 2006; Burnham & Hare, 2007; Burton-Chellew, El Mouden, & West, 2016; Burton-Chellew, Nax, & West, 2015; Burton-Chellew & West, 2013, 2021; Hagen & Hammerstein, 2006; Haley & Fessler, 2005; Krupp et al., 2005; McAuliffe, Burton-Chellew, & McCullough, 2019; Pedersen, Kurzban, & McCullough, 2013; Raihani & Bshary, 2015; Silva & Mace, 2014).

Despite decades of study there is still much debate and controversy over these competing explanations (Burton-Chellew & West, 2021; Camerer, 2013; Fehr & Schurtenberger, 2018). One reason for this is because the standard experimental design typically confounds these two explanations, by coupling together information on earnings from the game (payoff information) with information on groupmates’ decisions (social information). If individuals can observe both payoff and social information, it is unclear which information is more psychologically salient and motivating behaviour. This is a problem because the two forms of information are often highly correlated: more cooperative groupmates lead to higher payoffs and vice versa. Consequently, both the Conditional Cooperators and the Confused Learners hypotheses can predict an increase in cooperation in response to more cooperation by others and vice versa (Burton-Chellew, El Mouden, & West, 2017b; Burton-Chellew et al., 2015; Nax, Burton-Chellew, West, & Young, 2016).

One solution to the problem of confounding is to engineer modified versions of the public-goods game that test for either Conditional Cooperators or Confused Learners (Andreoni, 1995; Andreozzi, Ploner, & Saral, 2020; Angelovski, Di Cagno, Güth, Marazzi, & Panaccione, 2018; Bayer, Renner, & Sausgruber, 2013; Burton-Chellew et al., 2016; Burton-Chellew & West, 2013; Croson, Fatas, & Neugebauer, 2005; Di Cagno, Galliera, Güth, & Panaccione, 2016; Ferraro & Vossler, 2010; Fischbacher & Gachter, 2010; Fischbacher et al., 2001; Gunnthorsdottir, Houser, & McCabe, 2007; Houser & Kurzban, 2002; Kocher, Martinsson, Persson, & Wang, 2016; Kuemmerli, Burton-Chellew, Ross-Gillespie, & West, 2010; Kurzban & Houser, 2005; Shapiro, 2009; Thoni & Volk, 2018). For example, the strategy method, which forces individuals to specify in advance how they will respond to their groupmates’ contributions, was used to show that many individuals will, if so prompted, condition their contributions on the contribution of their groupmates, consistent with a concern for equality (Fischbacher & Gachter, 2010; Fischbacher et al., 2001; Kocher, Cherry, Kroll, Netzer, & Sutter, 2008). Alternatively, games played with computerised groupmates have also shown conditional behaviour, symptomatic of ‘confusion’, and games with a ‘black box’ have shown similar declines in contributions that can only be explained by payoff-based learning (Burton-Chellew et al., 2016; Burton-Chellew & West, 2013, 2021; Ferraro & Vossler, 2010; Houser & Kurzban, 2002; Shapiro, 2009). However, while these experiments are informative, it could be argued these modified public-goods games are problematic because they are either not the same game or not the same decision mechanism, and thus behaviour may change in unknown ways (Camerer, 2013; Fischbacher, Gächter, & Quercia, 2012).

**Figure 1.**
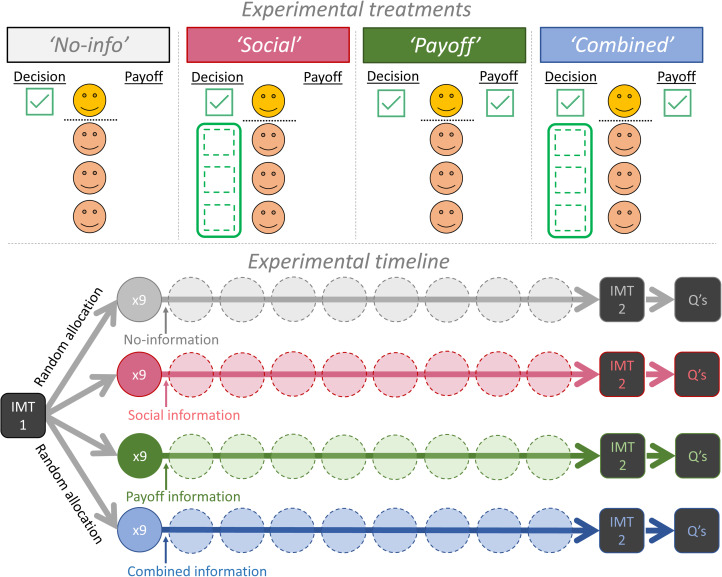
Experimental design. We decoupled two typical forms of information, about either the contribution decisions of the focal player's groupmates (social information) or the focal individual's own earnings (yellow/top individual, payoff information). This made four general treatments: one treatment with neither form of information (‘No-info’, *N =* 21 groups); one with just social information (‘Social’, *N =* 53 groups; 20 groups observed all individual decisions whereas 33 groups observed the group average only); one with just payoff information (‘Payoff’, *N =* 40 groups); and one with both forms of information (‘Combined’, *N =* 40 groups; 20/20 groups with either full/limited social information). We never showed the earnings (payoff) of groupmates. Experimental timeline: participants first had one round of decision making with computerised groupmates in a public-goods game (income maximisation test, IMT1). They were then randomly assigned to one of four information treatments for nine rounds in a constant group of four real people. They then repeated the income maximisation test (IMT2) before two survey questions (Qs) regarding their motivation and their understanding of the payoffs.

Here we avoided this potential problem by retaining the basic game and decision process of the standard public-goods game. We used standard instructions but merely varied what information participants received as feedback after each round of decision making (Fischbacher & Gachter, 2010). This way we could decouple payoff information from social information during the learning process (Figure 1). Our primary method was to provide individuals with either just their personal payoff information (Payoff treatment), or just social information on the decisions of their groupmates (Social treatment, individual decisions were shown in either full, or just the group average decision). This decoupling allowed us to evaluate the relative importance of payoff-based learning and conditional cooperation for causing the decline in contributions. If the decline is only due to payoff-based learning, then the decline will not occur unless payoff information is shown i.e., when participants are only shown social information. In contrast, if the decline is only due to conditional cooperation, then adding payoff information will not increase the rate of decline.

We also included a control treatment to provide a baseline measure for how behaviour may change over time when individuals have neither payoff nor social information, but still have time to reflect and re-read the payoff instructions (No-info treatment, Figure 1; Burton-Chellew et al., 2017b; Neugebauer et al., 2009). We also included a fourth treatment that presented the usual, combined, information, replicating the typical design in the literature (Combined treatment). Crucially, this combined treatment allowed us to evaluate the effect of adding payoff information to social information, which is technically redundant if individuals fully understand the game (a key assumption of the conditional cooperation interpretation). This redundancy is a strength of our design, because if individuals perfectly understand the game, then behaviour in the Combined treatment should not differ from the Social treatment. If, on the other hand, individuals are confused learners, their behaviour should differ across treatments, with a faster decline in contributions when payoffs are shown. We also included a range of alternative decision tasks both before and after the main game to test for confusion and motivations more directly (Figure 1).

## Methods

### Data collection

We collected data two times, a year apart. The collection bouts were identical in design except that degree of social information showed either all individual contributions (full social information), or just the group average contribution (limited social information). In both cases adding payoff information is technically redundant because both forms can be used to calculate personal payoffs, but only the limited social information can be calculated from personal payoffs. The first collection (‘study 1’) used detailed social information and 280 participants (70 groups) in the Autumn semester of 2018, from 21 to 30 November. The second bout of data collection (‘study 2’), with limited social information, used 336 participants (84 groups) in the Autumn semester of 2019, from 7 to 11 October. All data were anonymous and collected electronically.

### Participants and location

In total we ran 40 sessions with 616 participants (154 groups). Each session involved 12–20 participants (three to five groups). All sessions were conducted at the Faculty of Business and Economics (HEC), University of Lausanne (UNIL), Switzerland, in the HEC-LABEX facility. LABEX forbids deceiving participants and requires that all experimental designs obtain prior ethical approval from the LABEX ethics committee. HEC-LABEX used the ORSEE software to recruit participants and excluded all participants from previous experiments by the same authors (Greiner, 2015). Participants were mostly students enrolled at either UNIL or the Swiss Federal Polytechnic School, and they can be from diverse ethnic, socio-economic and cultural backgrounds (these variables were not recorded). According to the questionnaires we had a near equal gender ratio (306 female, 304 male, 2 other, and 4 declined to answer) and most of our participants were under 26 years of age (277 aged under 20, 309 aged 20–25, 25 aged 26–30, two aged 30–35, two over 35 and one declined to answer, responses to the age question were categorical to increase anonymity). We wanted to avoid participants that had studied the game theory of social dilemmas so in study 1 we excluded HEC students. In study 2 we were advised to allow first year students as it was early in the academic year.

### Financial incentives

We paid all participants a show-up fee of 10 Swiss Francs (CHF). For each of the 11 incentivised decisions, we endowed participants with 20 Monetary Units (MU) and each MU was worth 0.025 CHF, so 20 MU were worth 0.5 CHF. While these stakes are not large, they are comparable with related literature, and generally speaking, studies have found little effect of using larger stakes (Kocher, Martinsson, & Visser, 2008; Larney, Rotella, & Barclay, 2019; Yamagishi, Li, Matsumoto, & Kiyonari, 2016).

### Experimental procedure

The experiment had several stages (Figure 1). We provide a full copy of the instructions in English in our Supplementary Methods. For each stage participants had to answer questions regarding their understanding of the instructions (‘control questions’). For all questions, participants were allowed unlimited time and two attempts before we showed them the correct answers.

Procedures were standard for a behavioural economics experiment. Participants were randomly assigned to a partitioned computer terminal. We announced that communication was forbidden, and that the HEC-LABEX forbids deception. Participants then progressed, at their own pace, through on-screen instructions and control questions based on hypothetical scenarios detailing the general public-goods game decision and payoffs. We used publicly available instructions, control questions, and structural parameters and translated them into French (Fischbacher & Gachter, 2010). The specific parameters were group sizes of four participants, an endowment of 20 MU, and a multiplier/efficiency Factor of 1.6, which meant all contributions were multiplied by 1.6 before being shared out equally, to give a marginal per capita return of 0.4. As this is less than 1, the rational selfish income maximising decision in a single round is to contribute 0 MU. At this stage the instructions have only described the general decision task and the payoffs involved. The instructions so far have not mentioned game length, type of groupmates or information feedback.

We then used an asocial control treatment to test if individuals maximised their income when there were no social concerns (pre-game income maximisation test, IMT1). We informed participants that they would face the same decision but in ‘a special case’ with computerised groupmates programmed to play randomly. We assured them that this decision would not be seen by other participants, would have no future consequences, and no financial consequences for other participants but did have financial consequences for them. We made them answer five true or false statements about the situation they faced and provided them with the correct answers afterwards (Supplementary Methods). After their decision, we did not provide them with any immediate feedback about their earnings or the computer decisions, to prevent learning about the game before the information treatments. This income maximisation test allowed us to estimate how much individuals still contribute even when they can have no rational concern for fairness or helping others.

Next, participants learned that they would now face the same decision but with real people, as outlined in the original instructions. We informed them that they had been randomly grouped with three other participants for nine rounds of decision making. Although repeated games can favour some strategic cooperation, previous studies using constant groups have still observed the typical decline in contributions (Andreoni & Croson, 2008; Burton-Chellew & West, 2021), and backwards induction would mean that the selfish income maximising decision in a finite game of known length among rational individuals is still to contribute 0 MU (Ambrus & Pathak, 2011; Burton-Chellew, El Mouden, & West, 2017a; Dijkstra & van Assen, 2017; Kreps, Milgrom, Roberts, & Wilson, 1982; Krockow, Colman, & Pulford, 2016). We then explained to each group, according to its randomly assigned treatment, what type of information they would all be shown after each round. We assured them that all groupmembers would receive the same type of information. Again, we also made them answer five true or false statements about the situation they faced.

Specifically, we told participants, in the:

- No-info treatment, ‘You and everyone else in the group will not receive any information after each round. No participants will be able to know your investments at any time. Your earnings will not be shown to you each round, but you will receive the money at the end of the experiment’.
- Social treatment, ‘The information that each person will receive will only be the decision of each group member/average decision of the group [study 1/study 2]. Your earnings will not be shown to you each round, but you will receive the money at the end of the experiment’.
- Payoff treatment, ‘The information that each person will receive will only be their own earnings in each round. No participants will be able to know your investments at any time’.
- Combined treatment, ‘The information that each person will receive will only be their own earnings, and the decision of each group member/and the average decision of the group [study 1/ study 2]’.

We varied the detail of the social information because showing the individual decisions, as we did in study 1, could either impede the decline via individuals attempting to strategically signal their cooperative behaviour, or could, conversely, accelerate the decline if individuals responded more strongly to the presence of low/zero contributors than high/full contributors (Burton-Chellew et al., 2017a; Carpenter, 2004). Therefore study 2 used the simpler information of just showing the group average to avoid these effects.

We used fewer groups in our baseline measure, the No-info treatment (*N =* 21), because we wanted to devote more resources to the other treatments. This was also justified because the individuals are independent data points in the No-info treatment (because they cannot affect the behaviour of their groupmates). We used more groups in the Social treatment (*N =* 53) because we reasoned it was the treatment that was least well studied in the literature (studies normally show participants their payoffs). Therefore, whenever we had enough participants to form more than four groups in a session (in ‘study 2’), we allocated the extra group to the Social treatment. Our sample sizes, of 20–53 groups per treatment, are comparable with if not larger than related literature (Andreoni, 1995; Bayer et al., 2013; Chaudhuri, Paichayontvijit, & Smith, 2017; Fischbacher & Gachter, 2010).

After the repeated public-goods game with information treatments the participants were then told that they would again face the special case with computers, for just one round. This post-game income maximisation test (IMT2) allowed us to measure how the different information treatments had affected learning about how to maximise income.

At the end of the experiment, we asked participants two unincentivised questions regarding their understanding of the game's payoffs, and their motivation when grouped with humans. Specifically, to test understanding of the game's payoffs, we asked them, ‘In the basic decision situation, played for one round only, if a player wants to maximise his or her earnings, should they decide their contribution depending on what the other people in their group contribute?’ Participants had a choice of four answers: the correct answer, ‘No’; or two incorrect answers, ‘Yes’ or ‘Sometimes’; or they could respond ‘Do not know’ (Burton-Chellew et al., 2016). Asking participants about a strictly one-shot game allowed us to more cleanly measure their understanding of the game's payoffs and the social dilemma involved than if we had asked them about a repeated game. If a participant does not know the correct answer to this question, then they do not understand the game's payoffs.

To measure motivations we asked, ‘Which of these descriptions best describes your motivation during the rounds with humans?’ and offered participants four possible responses, that corresponded to selfishness (‘Making myself as much money as possible’), competitiveness (‘Making myself more money than other people’), a desire for fairness, as is often assumed to motivate conditional cooperation (‘Avoiding unequal outcomes so that I make neither more, nor less, than other people’) (Fehr & Fischbacher, 2003; Fehr & Schmidt, 1999; Fehr & Schurtenberger, 2018) or a desire to help the group (‘Making the group as much money as possible even if it meant making myself less money’). For both questions we reversed the on-screen order of responses for half of the participants (two within each group).

### Statistical analyses

Our analysis script is available on the Open Science Framework. We analysed the data using R-Studio version 1.3.1093 (R Team, 2020). Data were imported with the zTree package (Kirchkamp, 2019). All statistical tests were two-tailed. Significance values in linear mixed models were estimated by the lmerTest package, which uses the Satterthwaite approximation for estimating degrees of freedom (Kuznetsova, Brockhoff, & Christensen, 2017). Data figures were made with the ggplot2 package (Wickham, 2009).

When analysing the rate of decline in contributions we did not use individual contributions because responses within constant groups are not independent data. Instead, we analysed mean group contributions, which also have the benefit of tending towards a normal distribution (central limit theorem, Crawley, 2007). This allowed us to use a linear mixed model (LMM), with more interpretable coefficients on the rate of decline. We used maximum likelihood rather than restricted maximum likelihood that specified group identity as a random effect (random intercept and slope for each group). The game round variable was modified by −1 for each round to set the intercept to equal round one. We compared a series of models using a likelihood ratio test to settle on an optimal model for the main analyses (Supplementary Table S1). The optimal model controlled for differences in average levels of contribution in study 1 and study 2 but did not model any interactions between year of study and treatment effects.

## Results

### Payoff information led to a greater decline than social information

**Figure 2.**
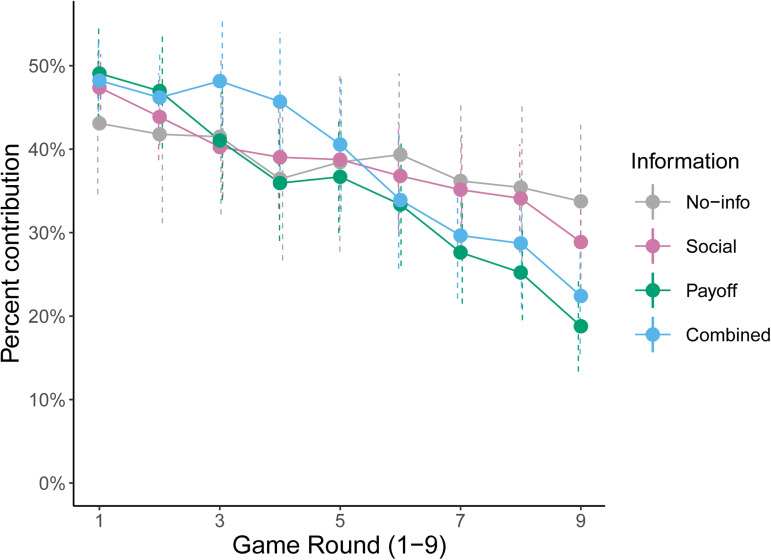
Declining cooperation. Depending on randomly assigned treatment, groups of individuals were shown no information (grey, 21 groups); or social information (magenta, 53 groups); or payoff information (green, 40 groups); or both social and payoff information combined (blue, 40 groups). Dashed vertical lines represent 95% confidence intervals for each round. Payoff information led to a greater decline than social information. Supplementary Figure S1 also shows the same data depending on the level of social information shown (either all individual decisions or just the group average).

In support of the Confused Learners hypothesis, contributions declined faster when we showed individuals their payoff information instead of social information (Figure 2). We found that the rate of decline was significantly faster among those shown only their payoff compared to those shown just social information (LMM, controlling for study: estimated difference in rate of decline between Payoff and Social treatment = −1.7 percentage points faster per round in the Payoff treatment, 95% CI = [−0.60, −2.73], *t*_1,154.0_ = −3.1, *p* = 0.002, Table 1). Specifically, when individuals saw their payoffs, contributions declined by −3.6 percentage points per round, decreasing from 49 to 18% over nine rounds, whereas among those shown only social information, contributions only declined at a rate of −1.9 percentage points per round, from 47 to 29% (Table 1). These rates are comparable with those found in a large comparative study of 237 games, which found an overall average rate of decline of 2.4 percentage points per round (although note that the rate depends on group size and marginal per capita return; Burton-Chellew & West, 2021).

**Table 1.** Declining cooperation**.** Results from a linear mixed model fit by maximum likelihood on the percentage contribution by each group per round depending on information treatment (reference treatment: social)

Fixed effects	Coefficient	SE	*t*^a^	d.f.	*p*-Value	Significance
(Intercept) [Social]	51.93	3.125	16.6	165.9	<0.001	***
Game round [Social]	–1.93	0.354	–5.4	154	<0.001	***
Year of study [2019]	–9.57	2.803	–3.4	154	<0.001	***
Treatment [No-info]	–4.20	4.874	–0.9	153.9	0.391	
Treatment [Payoff]	2.21	3.967	0.6	154.2	0.579	
Treatment [Combined]	4.66	3.967	1.2	154.2	0.242	
Game round × Treatment [No-info]	0.86	0.665	1.3	154	0.197	
Game round × Treatment [Payoff]	–1.67	0.54	–3.1	154	0.002	**
Game round × Treatment [Combined]	−1.48	0.54	–2.7	154	0.007	**
Random effects	Variance	Standard deviation				
Group ID (intercept)	346.4	18.61				
Group ID (game round)	4.88	2.21				
Residual	106.6	10.33				

Number of observations = 1386.

Number of independent groups = 154.

a *t*-Tests use Satterthwaite's method for estimating degrees of freedom.

### Social information had a negligible effect

In contrast, the effect of showing individuals social information had a negligible effect upon the rate of decline, despite being central to the Conditional Cooperators hypothesis. Compared with those individuals who saw no information (No-info treatment), the rate of decline was not significantly faster when individuals saw only information on the decisions of their groupmates, strongly contradicting the Conditional Cooperators hypothesis (Social treatment) (LMM: estimated difference in rate of decline between No-info and Social treatments = −0.9 percentage points per round, 95% CI = [ 2.17, −0.45], *t*_1,154.0_ = −1.3, *p* = 0.197, Figure 2, Table 1). These results were robust to an alternative analysis using a generalised linear mixed model to control for a potential lack of normality in the distribution of the response variable (Supplementary Results, Supplementary Table S2).

Repeating the above analyses separately for each study, which had different levels of social information, did not qualitatively change the conclusion that payoff information is what drives the decline (Supplementary Results, Supplementary Figure S1, Supplementary Tables S3 and S4). There was also no overall significant three-way interaction between study, game round and information treatment (LMM: Study × Game round × Information treatment, *F*_3,154_ = 0.7, *p* = 0.575, Supplementary Table S1).

### Redundant payoff information accelerated the decline

We confirmed our main result, that the rate of decline was faster when payoff information was available, with alternative analyses. First, we found that the addition of social information to payoff information did not lead to a significantly faster decline in the Combined information treatment compared to the Payoff treatment (LMM: estimated difference in rate of decline between Payoff and Combined treatments = 0.2 percentage points per round, 95% CI = [1.33, −0.95], *t*_1,154.0_ = 0.3, *p* = 0.744). This was either because individuals were learning from their payoffs in both cases or because individuals without social information were using their payoffs to calculate their groupmates’ decisions before responding conditionally.

However, if individuals were using their payoffs to calculate their groupmates’ decisions, then the rate of decline should not have been faster when the technically redundant payoff information was added to social information, but this is what we found. Specifically, the rate of decline was significantly faster among those shown both social and payoff information (Combined information treatment), than among those shown just social information (Social treatment, LMM: estimated difference in rate of decline between Social and Combined treatments = −1.5 percentage points faster per round in the Combined treatment, 95% CI = [−0.41, −2.54], *t*_1,154.0_ = −2.7, *p* = 0.007). This means that the addition of payoff information to social information led to a faster decline, despite this information being technically redundant, in support of the Confused Learners hypothesis and contradicting the Conditional Cooperators hypothesis.

Second, for descriptive purposes, we analysed all the groups separately (Table 2), and found that groups which saw payoff information were almost twice as likely to develop a negative correlation between their contributions and round of the game (percentage of groups with a significant negative Pearson correlation, Payoff treatment = 70%; Combined treatment = 65%; Social treatment = 36%, No-info treatment = 24%; total number of groups = 40/40/53/21 respectively). Among those groups in the Social treatment, 17% even finished at a higher level than their starting level (*N =* 9/53). In contrast, only 2% of groups in the Payoff treatment finished at a higher level (*N =* 1/40) (Supplementary Figures S2–S5 show the time profiles for each group). These comparisons would suggest that the Social treatment, even though it implicitly contained payoff information, was behaviourally more akin to the No-info treatment than to the two treatments showing explicit payoff information (Payoff and Combined treatments).

**Table 2.** Group summaries. Shown per treatment are the percentage of groups that finished the final round either lower or higher than their first round, and the percentage that had a significant Pearson correlation between contributions and all nine rounds of the game

Treatment (*N*)	Lower	Higher	Significantly negative	Significantly positive
No-info (21)	76%	24%	24%	0%
Social (53)	83%	17%	36%	4%
Payoff (40)	98%	2%	70%	0%
Combined (40)	90%	10%	65%	5%

### Supplementary tasks support the Confused Learners hypothesis

The results from the dynamic behaviour in the repeated public-goods game found that payoff information, and not social information, drove the decline in contributions. Next, we analysed the results from a range of supplementary decision tasks to test for understanding and motivations more directly (Figure 1). Our results from these three tasks confirmed that most participants are self-interested but confused.

### First, most participants failed to maximise their income in the asocial control

**Figure 3.**
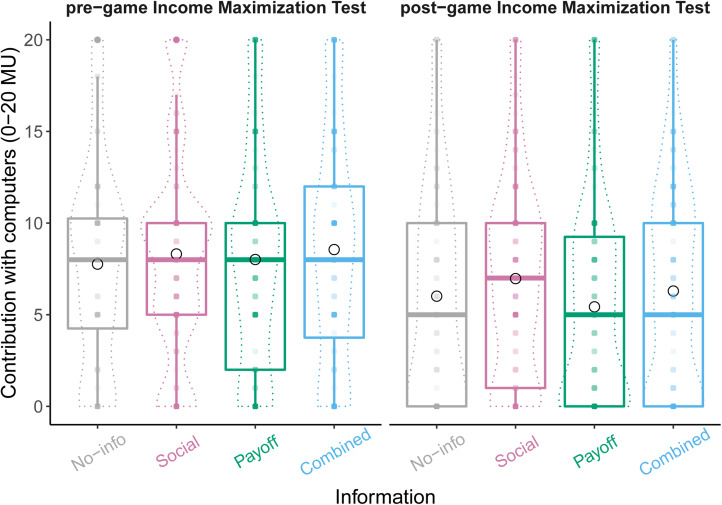
Income maximisation tests. Most individuals failed to maximise their income even when grouped with computers. Violin plots show distribution of contributions. Box plots show the median contribution (horizontal bar) and interquartile range. The mean contributions are shown by the empty black circles. We tested all individuals twice, one before and once after the repeated public-goods game. The least improvement was among individuals shown only social information (pink). Number of individuals: No-info = 84; Social = 212; Payoff =160; Combined = 160.

In support of the Confused Learners hypothesis, we found (1) that in the pre-game income maximisation test played with computers (IMT1), most participants (82%) contributed and thus behaved as if confused. This was irrational because they merely ‘burnt’ money for no social benefit (82% contributed more than 0 MU, *N =* 504/616; mean average contribution = 41%, or 8.2/20 MU, median = 8 MU); and (2) that the greatest improvement in the post-game test (IMT2) occurred among those who had experienced the treatments containing payoff information (Payoff and Combined treatments vs Social and No-info treatments, generalised linear quasibinomial model controlling for individual's contribution in the pre-game test: *t*_1,612_ = −2.6, *p* = 0.009, Figure 3, Supplementary Figure S6, Supplementary Table S5).

If the decline in contributions in the repeated games was caused by Conditional Cooperators changing their beliefs about their groupmates, then behaviour towards computers programmed to play randomly should not have changed between the pre- and post-game income maximisation tests (IMT1 vs IMT2), but that is not what we found. Instead, we found that improvement in the income maximisation tests, where contributions decreased from 41% in IMT1 to 27–35% in IMT2 depending on treatment, tracked changes in the repeated game with humans (where contributions decreased from 47 to 19–34% depending on treatment), suggesting learning about the game's payoffs was responsible. The surprising result was that there was a significant improvement in the No-info condition (paired *t*-test, mean of difference = 1.75 MU (9%), 95% CI = [0.41 MU, 3.09 MU], *t* = 2.6, d.f. = 83, p = 0.011), perhaps because the participants used their time to reflect and re-read the instructions which were always available in a help box.

### Second, most participants appeared confused about the nature of the payoffs

Participants appeared to believe the game's payoffs were interdependent, meaning one needs to know the behaviour of others to calculate the best income maximising response, which is not true in the linear public-goods game. Specifically, when we asked participants if the income-maximising strategy depended on the actions of their groupmates or not, only 25% of participants answered correctly (‘No’) (*N* = 153/616). In contrast, 72% of participant answered incorrectly (*N* = 444), indicating that they had false beliefs about the game's Payoff, with 53% responding that ‘Yes’, an income maximising individual should decide their contribution depending on what others contribute (*N* = 325) and another 19% responding ‘Sometimes’ (*N* = 119). The remaining participants, 3%, indicated that they did not know (‘Do not know’, *N =* 19) (Table 3). Incorrect individuals were significantly more likely to have cooperated with computers, consistent with confused conditional cooperation (Burton-Chellew et al., 2016; numbers that contributed more than 0 MU towards computers: in IMT1, *N =* 399 of 463 non-correct responses vs 105 of 153 correct responses; Fisher's exact test, *p* < 0.001; in IMT2, *N =* 358/463 vs 75/153; Fisher's exact test, *p* < 0.001).

**Table 3.** Confusion about payoffs. Summary of participant responses to: ‘In the basic decision situation, played for one round only, if a player wants to maximise his or her earnings, should they decide their contribution depending on what the other people in their group contribute?’^a^

Information	‘Yes’	‘No’^b^	‘Sometimes’	‘Do not know’	*N*
No-info	54% (45)	25% (21)	20% (17)	1% (1)	84
Social	50% (105)	26% (55)	21% (45)	3% (7)	212
Payoff	55% (88)	26% (42)	15% (24)	4% (6)	160
Combined	54% (87)	22% (35)	21% (33)	3% (5)	160
Overall	53% (325)	25% (153)	19% (119)	3% (19)	616

a We reversed the order of responses for half of the participants (2 within each group).

b This is the correct response.

### Finally, most participants reported selfish motives

Clearly one important aim of laboratory experiments is to gain knowledge about the specific motives behind social behaviours (Fehr & Fischbacher, 2003). This is traditionally done with incentivised decisions, in the hope that the costs of decisions will inhibit non-genuine responses, and consequently reveal participants’ true motivations (Andreoni & Miller, 2002). However, such an economical/mathematical approach risks being confounded by greater misunderstanding. Therefore, we simply asked participants to identify with one of four possible motivations, which should provide a lower bound estimate on the level of non-socially describable motivations (Methods, Table 4). The results contradicted the idea that most people are fair-minded conditional cooperators. Instead, most participants, 54%, responded that they were best described by the selfish motivation (*N =* 305/568 surveyed). A further 6% responded that they had a competitive motivation (*N =* 35), meaning that 60% of participants freely admitted to a socially undesirable response (*N =* 340). In contrast, only 28% responded that they were best described by the fair or inequity-averse motivation (*N =* 160) and only 12% that they were motivated by maximising the group's success even at personal cost (pro-group motivation) (*N* = 68) (Table 4).

**Table 4.** Selfish motivations. Summary of participant responses to: which of these descriptions best describes your motivation during the rounds with humans?^a^

Information	Selfish^b^	Inequity-averse^c^	Pro-group^d^	Competitive^e^	*N*^f^
No-info	57% (41)	24% (17)	11% (8)	8% (6)	72
Social	51.5% (103)	29% (58)	14.5% (29)	5% (10)	200
Payoff	59% (87)	27% (40)	9% (13)	5% (8)	148
Combined	50% (74)	30% (45)	12% (18)	7% (11)	148
Overall	54% (305)	28% (160)	12% (68)	6% (35)	568

a We reversed the order of responses for half of the participants (two within each group).

b ‘Making myself as much money as possible’.

c ‘Avoiding unequal outcomes so that I make neither more, nor less, than other people’.

d ‘Making the group as much money as possible even if it meant making myself less money’.

e ‘Making myself more money than other people.’

f Forty-eight participants, 12 in each treatment, were not presented with this question.

## Discussion

We tested competing explanations for behaviour in public-goods games by controlling whether individuals could learn from their own payoffs or not (Confused Learner's hypothesis), and whether they could respond conditionally to their groupmates’ contributions or not (Conditional Cooperators hypothesis; Figure 1). We found substantial support for the Confused Learner's hypothesis across multiple results. Specifically, we found that: (1) payoff information generated the greatest decline in contributions, regardless of whether social information was present or not (Figure 2, Tables 1 and 2); (2) most individuals demonstrated confusion about the game's payoffs when playing with computerised groupmates (Figure 3) or when directly asked (Table 3); and (3) that most individuals admitted to being selfishly motivated to make themselves as much money as possible (Table 4). If more than 50% of participants freely admit to being selfishly motivated, it seems unjustified to conclude that humans are especially altruistic.

Overall, our results suggest that apparent altruism in public-goods games mostly arises from confused but self-interested individuals trying to learn how to play the game, and not from fair-minded altruistic individuals trying to help their group or equalise payoffs. If individuals are confused, we cannot be sure what game they think they are playing (Chou, McConnell, Nagel, & Plott, 2009; Columbus, Munich, & Gerpott, 2020; Ferraro & Vossler, 2010; Plott & Zeiler, 2005). For example, if individuals erroneously think the best action depends on what others do, like a ‘stag-hunt’ game or ‘threshold’ public-goods game, perhaps because these games are more common in daily life, then it makes sense for them to act conditionally on social information even if they are self-interested (Croson & Marks, 2000; Henrich et al., 2005; Rondeau, Poe, & Schulze, 2005). This could explain why individuals appear to conditionally cooperate with computers, that cannot benefit, and why responses to our question on how to maximise incomes closely resembled the frequencies of different ‘social types’ typically reported in other experiments (~50–55% = ‘Yes’ = ‘Conditional Cooperator’; ~20–30% = ‘No’ = ‘Free Rider’, etc.; Burton-Chellew et al., 2016; Fischbacher & Gachter, 2010; Fischbacher et al., 2001; Thoni & Volk, 2018). In this case social types would really be artefacts of variation in levels of understanding (Burton-Chellew et al., 2016) and repeated public-goods games with payoff information would be measuring rates of learning rather than social preferences (Burton-Chellew & West, 2021). This is consistent with the recent finding that the apparent preference for conditional cooperation decreases with experience, contradicting a key assumption of the Conditional Cooperators hypothesis (Andreozzi et al., 2020).

Our study shows the value of using a range of treatments and methods. For example, our No-info treatment provided an informative baseline treatment when comparing rates of decline (Supplementary Tables S3 and S4). Our simple, direct, surveys of understanding and motivation complimented our use of the more traditional methods of incentivised decision making (‘behavioural economics’). The latter is less susceptible to socially desirable responses, but the former is simpler to understand and the results easier to interpret. Our income maximisation tests with computerised groupmates provided quick and simple diagnostic tests of ‘rational’ behaviour, a commonly assumed hypothesis in economic experiments aiming to measure social behaviours.

### Future directions

It could be useful to expand our experiment. One could test which information is of more interest to participants by allowing them to choose (Burton-Chellew & D'Amico, 2021), or to use eye or mouse tracking software (Geran & Weixing, 2020; Jiang, Potters, & Funaki, 2016; Lahey & Oxley, 2016). One could vary how groups are formed, group size, or the length of the game, to investigate if the salience of social information depends on the probability of future social interactions (Burton-Chellew et al., 2017a; Fiala & Suetens, 2017; Reuben & Suetens, 2012; Trivers, 2006). One could ask participants about their expectations about their groupmates’ contributions to gain insights into their thought processes (Chaudhuri et al., 2017; Fischbacher & Gachter, 2010). We did not do so because asking individuals to guess the contributions of their groupmates may distort behaviour by stimulating conditional cooperation in individuals that would otherwise not have thought about their groupmates. One could use a range of different instructions to test if our results generalise to different set-ups or different cultures (Li, 2017). Here we simply replicated common instructions and used a common participant pool, to enable comparisons with many prior key studies (Fehr & Gachter, 2002; Fischbacher & Gachter, 2010; Fischbacher et al., 2001). We replicated the standard result, but our expanded design and varied approach allowed us to identify that most participants were in fact self-interested, not altruistic, but needed to learn about the game's payoffs.

## Data Availability

The data and analyses that support the findings of this study are openly available in the Open Science Framework at https://osf.io/t4smj

## Supplementary material

For supplementary material accompanying this paper visit https://doi.org/10.1017/ehs.2022.45. click here to view supplementary material
