# Supplementary material for: Self-interested learning is more important than fair-minded conditional cooperation in public-goods games
Source: Evol Hum Sci. 2022 Oct 17;4:e46. doi: 10.1017/ehs.2022.45 (PMC10426038; doi:10.1017/ehs.2022.45)
Supplement: Supplementary file 1 [file S2513843X22000457sup001.pdf]

SUPPLEMENTARY INFORMATION for:

Self-interested learning is more important than fair-minded conditional cooperation in public-goods games

Maxwell N. Burton-Chellew*[1,2] & Claire Guérin[2]

*Corresponding author: maxwell.burton@unil.ch

[1]Department of Economics, HEC-University of Lausanne, 1015 Lausanne, Switzerland

[2]Department of Ecology and Evolution, Biophore, University of Lausanne, 1015 Lausanne, Switzerland.

Version date: Tuesday 4th October 2022

Contents

Supplementary Methods

Supplementary Results

Supplementary Figures (1-6)

Supplementary Tables (1-5)

SUPPLEMENTARY METHODS

A full copy of the instructions translated into English are shown at the end of these Supplementary Methods. The data and analyses that support the findings of this study are openly available in the Open Science Framework at https://osf.io/t4smj

Copy of instructions

Here is a copy of the instructions, which were translated into French for our participants. The instructions for the general game were copied as much as possible from reference (U. Fischbacher & Gachter, 2010)

Stage 1 screen 1 – general instructions

You are now taking part in an economic experiment, which has been financed purely for academic research purposes.
If you read the following instructions carefully, you can, depending on your decisions, earn a reasonable amount of money.
It is therefore very important that you read these instructions with care.

The instructions, which we have distributed to you, are solely for your private information.
You are not allowed to communicate during the experiment.
If you have any questions, please ask us. Violation of this rule will lead to your exclusion from the experiment and all payments.
If you have questions, please raise your hand. A member of the experimenter team will come to you and answer them in private.

During the experiment we shall not speak of CHF but rather of MU (Monetary Units).
During the experiment your entire earnings will be calculated in MU.
At the end of the experiment the total amount of MU you have earned will be converted to CHF at the following rate:
1 MU = 0.05 CHF, so 20 MU = 1 CHF and 100 MU = 5 CHF.
At the end of the experiment your entire earnings from the experiment will be immediately paid to you in cash.

We describe the experimental process below.

Stage 1 screen 2

The decision situation

You will learn how the experiment will be conducted later. We first introduce you to the basic decision situation. You will find control questions at the end of the instructions that help you to understand the decision situation.

You will be in a group consisting of **4 people**. Each group member has to decide on the allocation of 20 MU. You can put these 20 MU into your **private account** or you can invest

them **fully or partially** into a project. Each point you do not invest into the project, will automatically remain in your private account.

Your income from the private account:

You will earn one MU for each MU that you put into your private account.
For example, if you put 20 MU into your private account (and therefore do not invest into the project) your income will amount to exactly 20 MU out of your private account.
If you put 6 MU into your private account, your income from this account will be 6 MU.
No one except you earns something from your private account.

Stage 1 screen 3

Your income from the project:

Each member of the group will also benefit from the amount you invest in the project.
On the other hand, you will also gain from the other group members' investments.
The income for each group member will be determined as follows:

***Income from the project =** sum of all contributions x 0.4*

If, for example, the sum of all contributions to the project is 60 MU, then you and the other members of your group each earn 60 x 0.4 = 24 MU out of the project.
If four members of the group contribute a total of 10 MU to the project, you and the other members of your group each earn 10 x 0.4 = 4 MU.

Total income:

Your total income is the sum of your income from your private account and that from the project:

Income from your private account (= 20 - contribution to the project)
+ Income from the project (= 0.4 x sum of all contributions to the project)

= Your Total Income

Stage 1 screen 4 – Control Question 1/4, for all questions, participants were allowed unlimited time and two attempts before we showed them the correct answers

Please answer the following four control questions. They will help you to gain an understanding of the calculation of your income, which varies with your decision about how to distribute your 20 MU.

There is a calculator available.

To access the calculator, click on the image in the bottom right of your screen.

Please enter your answers into the spaces provided and press Continue when done.

Question 1: Each group member has an endowment of 20 MU. Nobody (including yourself) contributes any MU to the project.

What is *your* total income (in MU)? [answer box, correct answer is 20]

What is the total income of each of the *other* group members? [answer box, correct answer is 20]

Stage 1 screen 5

Here is the second question.

Question 2: Each group member has an endowment of 20 MU. You invest 20 MU in the project. Each of the other three members of the group also contributes 20 MU to the project.

What is *your* total income (in MU)? [answer box, correct answer is 32]

What is the total income of each of the *other* group members? [answer box, correct answer is 32]

Stage 1 screen 6

Here is the third question.

Question 3: Each group member has an endowment of 20 MU. The other three group members contribute a total of 30 MU to the project.

What is *your* total income (in MU), if you - in addition to the 30 MU - invest 0 MU into the project? [answer box, correct answer is 32]

What is *your* total income (in MU), if you - in addition to the 30 MU - invest 10 MU into the project? [answer box, correct answer is 26]

What is *your* total income (in MU), if you - in addition to the 30 MU - invest 15 MU into the project? [answer box, correct answer is 23]

Stage 1 screen 7

Here is the final question.

Question 4: Each group member has an endowment of 20 MU. Assume that you invest 8 MU to the project.

What is *your* total income (in MU) if the other group members together - in addition to your 8 MU - contribute another 7 MU in total to the project? [answer box, correct answer is 18]

What is *your* total income (in MU) if the other group members together - in addition to your 8 MU - contribute another 12 MU in total to the project? [answer box, correct answer is 20]

What is *your* total income (in MU) if the other group members together - in addition to your 8 MU - contribute another 22 MU in total to the project? [answer box, correct answer is 24]

Stage 2 screen 1 – pre-game Income Maximization Test (IMT1)

Before beginning, you are going to play this game in a **special case. You will face the same decision but in a special case.**

In this special case, **your income will be calculated in the same was previously described.**

However, in this special case, you will be in a group of just you and the COMPUTER.

The situation will be the same as described in the instructions,

but instead of 3 other people, it will just be you and **the computer.**

The computer will make the decisions of the other 3 players.

The computer will choose their decisions **randomly and separately** (so each computer player will make its own random decision).

You are the only person in the group, and only you will receive any money.

Nobody but the experimenter will know your decision.

You will now play this special case for only one round.

You will not be told what happened but will receive the money at the end of the experiment. After playing this special case, you will go on to play in groups of 4 people as described in the instructions.

Nobody but the experimenter will know your decision. Your decision will have no consequences for the rest of the experiment.

Stage 2 screen 2– control questions, we allowed two attempts then showed the correct answers (TRUE/FALSE) on the next screen

Please confirm your understanding of the previous instructions. Enter 1 if the statement is True, or 0 if the statement is False

You are in a group with Computers [answer box, correct answer is 1/TRUE]

You are in a group with Humans [answer box, correct answer is 0/FALSE]

People in your group will see your decision [answer box, correct answer is 0/FALSE]

You will see the decisions of people in your group [answer box, correct answer is 0/FALSE]

You will see your payoffs after each round [answer box, correct answer is 0/FALSE]

Stage 2 screen 3 – pre-game IMT contribution screen

Please enter your contribution (between 0 – 20)
Your contribution to the project is [entry box]

Help box

[Contained a full copy of the instructions regarding the decision and payoff mechanism]

Stage 2 screen 4 – pre-game IMT feedback

You received an endowment of 20 MU
You invested in the group project with computers [X] MU
Your final income from this round is being calculated

You will receive the information on your income at the end of the experiment. Please press continue to move onto the next round.

Stage 3 screen 1 – repeated public goods game with information treatments, certain elements of the instructions varied necessarily according to treatment.

You will now play in a group of 4 people as described in the instructions.

You have been put into a randomly formed group of 4 people.

You will play in this new group, with the same 3 other people, for 9 rounds of decision making.

[No-info treatment]
You and everyone else in the group will not receive any information after each round.
No participants will be able to know your investments at any time.
Your earnings will not be shown to you each round, but you will receive the money at the end of the experiment.

[all other information treatments]
You will receive some information after each round.
You and everybody else in your group will receive the same type of information.

[Social treatment, Study 1 with full social information]
The information that each person will receive will only be the decision of each group member.
Your earnings will not be shown to you each round, but you will receive the money at the end of the experiment.

[Social treatment, Study 2 with limited social information]
The information that each person will receive will only be the average decision of the group.
Your earnings will not be shown to you each round, but you will receive the money at the end of the experiment.

[Payoff treatment]

The information that each person will receive will only be their own earnings in each round. No participants will be able to know your investments at any time.

[Combined treatment, Study 2 with limited social information]
The information that each person will receive will only be their own earnings and the decision of each group member.

[Combined treatment, Study 2 with limited social information]
The information that each person will receive will only be their own earnings and the average decision of the group.

"*I understand I am now playing with real people*" [annotated continue button]

Stage 3 screen 2 – a repeat of the control questions from Stage 2 (IMT1); we allowed two attempts then showed the correct answers (TRUE/FALSE) on the next screen
Please confirm your understanding of the previous instructions. Enter 1 if the statement is True, or 0 if the statement is False

You are in a group with Computers [answer box, correct answer is 0/FALSE]

You are in a group with Humans [answer box, correct answer is 1/TRUE]

People in your group will see your decision [answer box, correct answer depends, shown in Study 1 only]

People in your group will see the average decision [answer box, correct answer depends, shown in Study 2 only]

You will see the decisions of people in your group [answer box, correct answer depends]

You will see your payoffs after each round [answer box, correct answer depends]

Stage 3 screen 3 – repeated public good game contribution screen

Round X of 9

Please enter your contribution (between 0 – 20)

Your contribution to the project is [entry box]

Help box

[Contained a full copy of the instructions regarding the decision and payoff mechanism]

Stage 3 screen 4 – feedback screens, [varied by treatment]

You received an endowment of 20 MU

You invested in the group project [X] MU

Member B invested [X] MU [Social and Combined treatments, study 1]

Member C invested [X] MU [Social and Combined treatments, study 1]

Member D invested [X] MU [Social and Combined treatments, study 1]

The average decision of the group was [X.Y] MU [Social and Combined treatments, study 2, average shown to 1 decimal place]

You received from the group account: [X] MU [Payoff and Combined treatments]

Your final earnings from this round are: [X] MU [Payoff and Combined treatments]

Your gain is: [X-20] MU [Payoff and Combined treatments]

Your earnings for this round are being calculated [No-info and Social treatments]

Stage 4 screen 1 – post-game Income Maximization Test (IMT2)

You will now make decisions again in the **special case from before with the COMPUTER**.

The decision situation will be the same as before,

but now instead of 3 other people, it will just be you and **the computer again**.

As before, the computer will again pick the decisions of the other 3 group members.

The computer will pick their decisions in the same way as before. That is, **randomly and separately** (so each computer 'member' will make its own random decision).

You are the only person in the group, and only you will receive any money.
You will now make this decision in this special case for only one round.
You will not be told what happened but will receive the money at the end of the experiment.

Nobody but the experimenter will know your decision. Your decision will have no consequences for the rest of the experiment.

Stage 4 screens 2-4 repeated the IMT process of control questions, contributing and feedback (Stage 2 screens 2-4)

SUPPLEMENTARY RESULTS

Robustness check

Our main analyses were based on a linear mixed model. We checked if the results were qualitatively the same with a generalized linear mixed model that accounted for the potential lack of a normal distribution in the response variable. We ran a model with identical fixed and random effects as in Table 1, but with a binomial logit link. All the results were qualitatively the same as in Table 1 (Supplementary Table 2). The rate of decline was significantly faster in both the Payoff treatment and in the Combined treatment compared to the Social treatment (GLMM: Payoff treatment, $z = -3.2$, $P = 0.002$; Combined treatment, $z = -2.6$, $P = 0.010$), but was not significantly different between the Social treatment and the No-info treatment (GLMM: $z = 1.4$, $P = 0.175$).

Separate analyses for each study

We found that, in both studies, the rate of decline in cooperation (contributions) significantly depended on the type of information shown (Linear mixed model ran separately for each study, with random intercept and slope for each group, LMM: Group

percentage contribution ~ Game round * Information treatment; Study 1, $F_{3,70.0}$ = 3.1, P = 0.033; Study 2, $F_{3,84.0}$ = 5.2, P = 0.002).

Repeating the analyses from the main Results, but separately for each study, we find that when individuals only saw their payoff and no other information (Payoff treatment), contributions declined at an estimated rate of -3.7 percentage points, 95% CI = [-2.44, -5.02] (Study 1) or -3.5 percentage points, 95% CI = [-2.48, -4.45] (Study 2), per round. This was significantly faster than those shown only detailed social information (Social treatment, Study 1) (LMM: estimated difference between Payoff and Social treatment, Study 1 = -1.7, 95% CI = [-0.57, -4.22], $t_{1,70.0}$ = 2.6, P = 0.011, Supplementary Table 2). However, the difference was not significant in Study 2 with the limited social information, although the estimate was in the same direction (LMM: estimated difference between Payoff and Social treatment, Study 2 = -1.2, 95% CI = [0.08, -2.42], $t_{1,84.0}$ = 1.9, P = 0.067, Supplementary Table 3).

Comparing the effect of adding different levels of social information (individual decisions versus just the group average) to no-information, we found that in both studies, the difference between the Social information treatment and the No-info treatment was non-significant. In study 1, with full social information (shown individual decisions), the estimated difference in the rate of decline was near zero (LMM: estimated difference = -0.03 percentage points per round, 95% CI = [2.20, -2.27], $t_{1,70.0}$ = -0.03, P = 0.974, Supplementary Table 2). In Study 2, with limited social information (the group average) there was nearly a significant increase in the decline (LMM: estimated difference = -1.4 percentage points, 95% CI = [0.11, -2.97], $t_{1,84.0}$ = -1.8, P = 0.069, Supplementary Table 3). Therefore it is unclear if the limited social information had an effect (Social treatment, Study 2), because although the slope was almost significantly steeper, the mean level of contribution in the final round was indistinguishable between those that had been shown limited social information, and those that been shown no information at all (Figure 2; linear model on final round group percent contributions: mean contribution in No-info treatment = 26.6%, 95% CI = [16.2, 37.0], estimated difference in Social treatment = -1.7% percentage points, 95% CI = [-13.79, 10.30], $t_{1,42}$ = -0.3, P = 0.772

Comparing rates of decline in the Combined information treatment with the Social and Payoff treatments. The rate of decline in the Combined information treatment which contained both social and payoff information, was significantly faster than in the Social treatment in Study 2 (limited social information) but no in Study 1 (full social information. Specifically, the estimated difference in rate of decline per round between Social and Combined treatments in Study 1 with full social information = -1.7 percentage points, 95% CI = [-3.53, 0.12], $t_{1,70.0}$ = 1.8, P = 0.067; and in Study 2 with limited social information = -1.5 percentage points, 95% CI = [-2.73, -0.23], $t_{1,84.0}$ =2.4, P = 0.021, Supplementary Tables 2-3).

However, the rate of decline in the Combined treatment was not significantly faster than in the Payoff treatment in either study (estimated difference in rate of decline per round between Payoff and Combined treatments in Study 1 with full social information = 0.7 percentage points per round, 95% CI = [2.51, -1.14], $t_{1,70.0}$ = 0.7, P = 0.456; in Study 2 with limited social information = -0.3 percentage points per round, 95% CI = [1.09, -1.71], $t_{1,84.0}$ = -0.4, P = 0.661, Supplementary Tables 2-3).

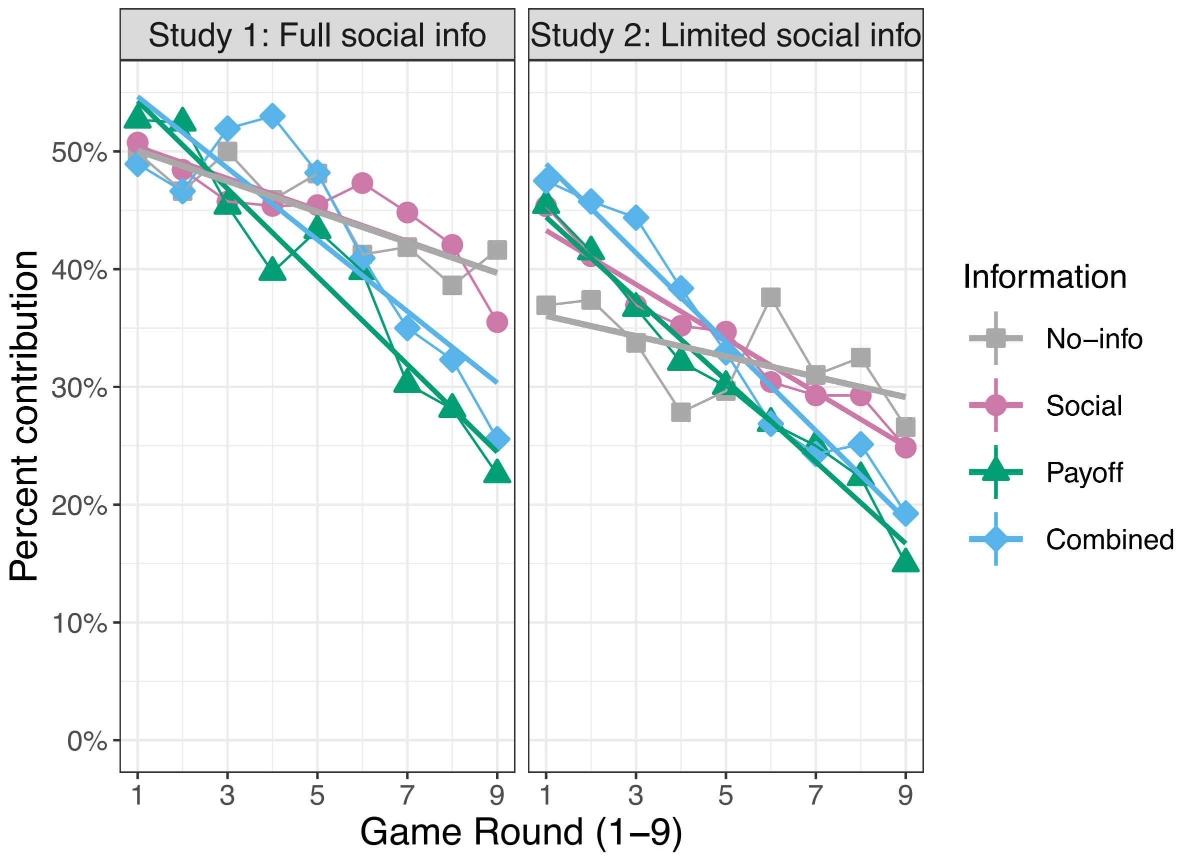

Supplementary Figure 1. Contributions over time for all treatments. This figure is for illustration purposes and the linear regressions shown do not consider random effects (the autocorrelation among groups).

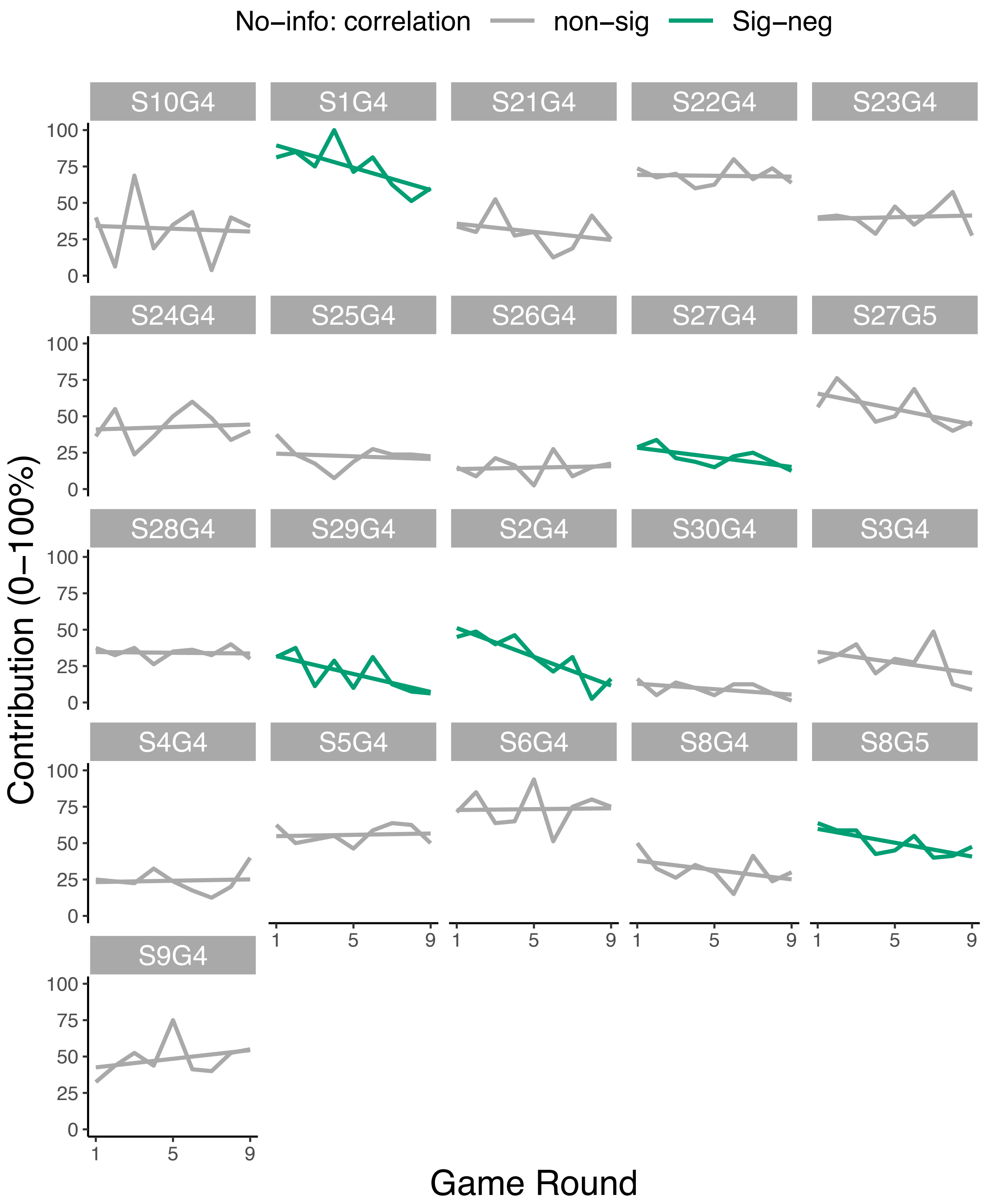

Supplementary Figure 2. Group profiles in the No-information treatment. Data show connecting line between the mean contributions per round and the estimated linear regression for each group, colour coded by if the Pearson correlation between game round and contribution was significant or not.

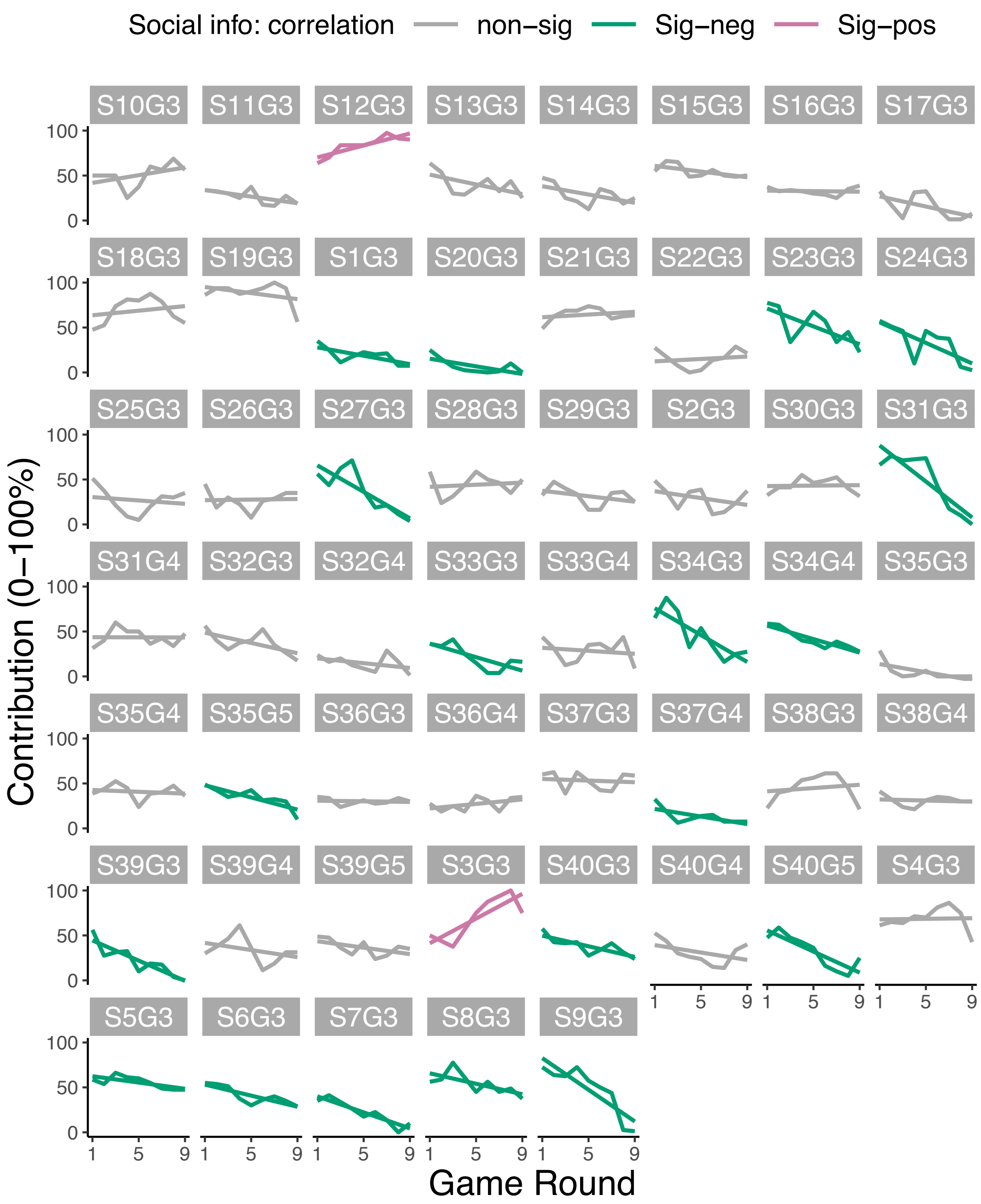

Supplementary Figure 3. Group profiles in the Social information treatment. Data show connecting line between the mean contributions per round and the estimated linear regression for each group, colour coded by if the Pearson correlation between game round and contribution was significant or not.

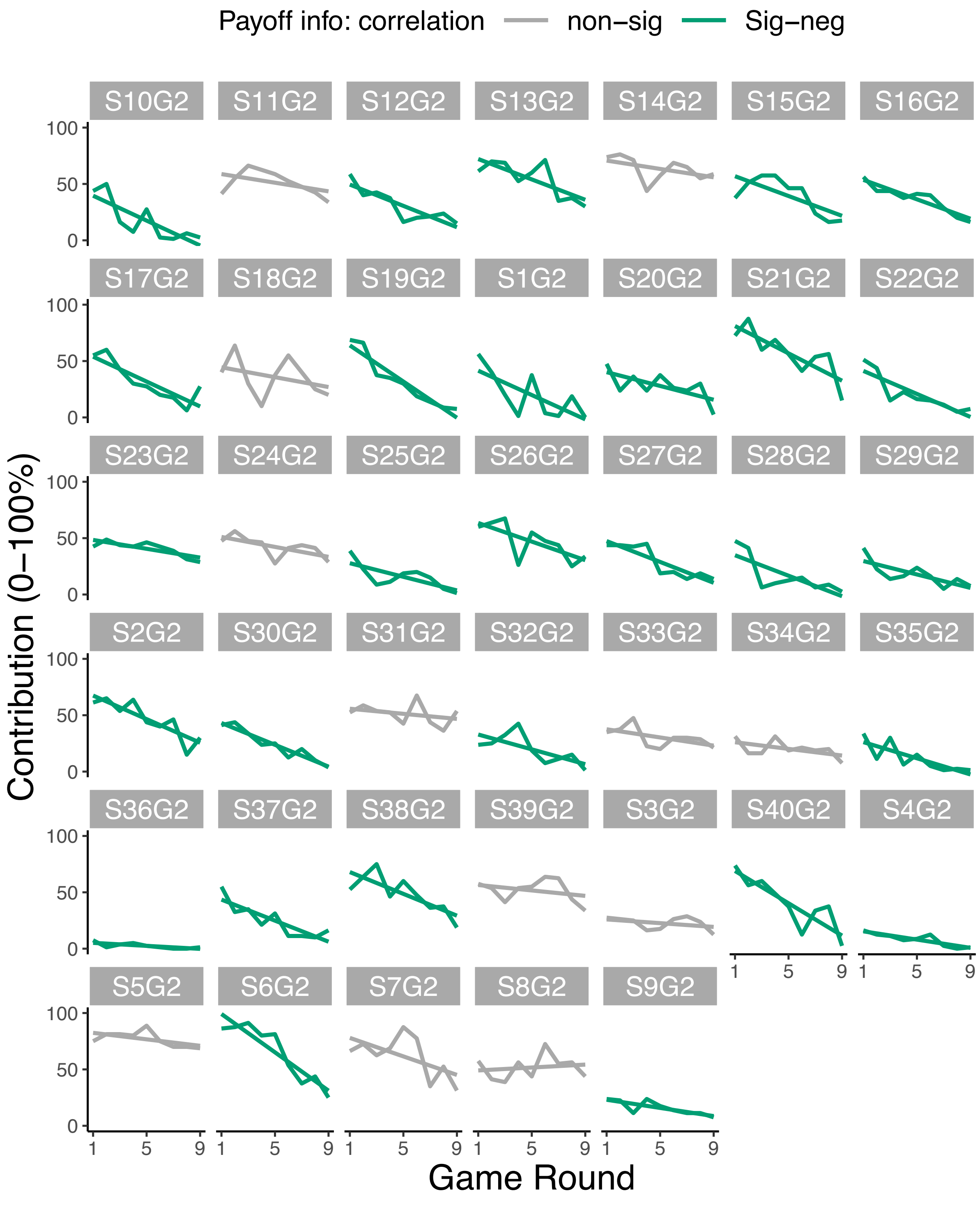

Supplementary Figure 4. Group profiles in the Payoff information treatment. Data show connecting line between the mean contributions per round and the estimated linear regression for each group, colour coded by if the Pearson correlation between game round and contribution was significant or not.

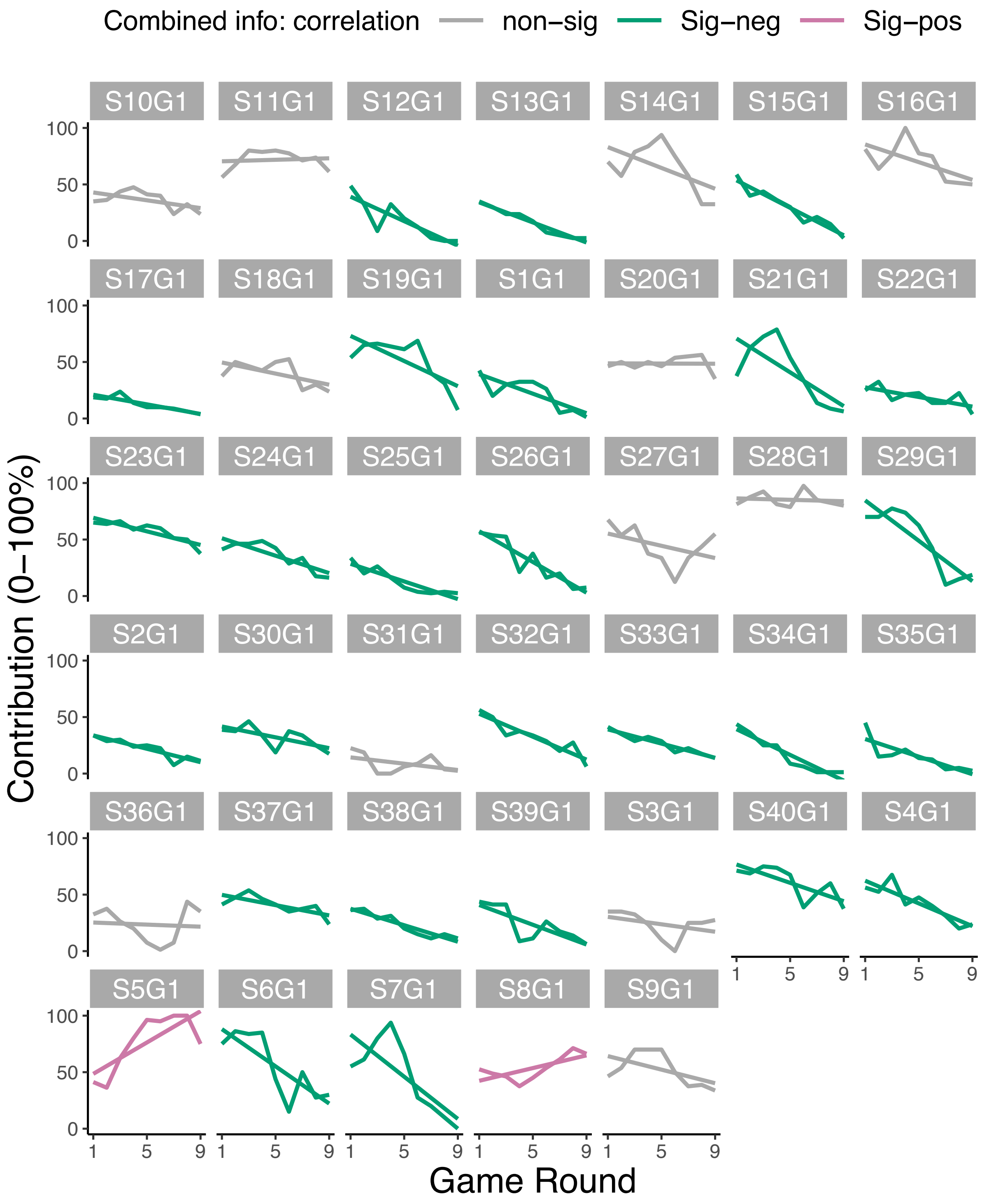

Supplementary Figure 5. Group profiles in the Combined information treatment. Data show connecting line between the mean contributions per round and the estimated linear regression for each group, colour coded by if the Pearson correlation between game round and contribution was significant or not.

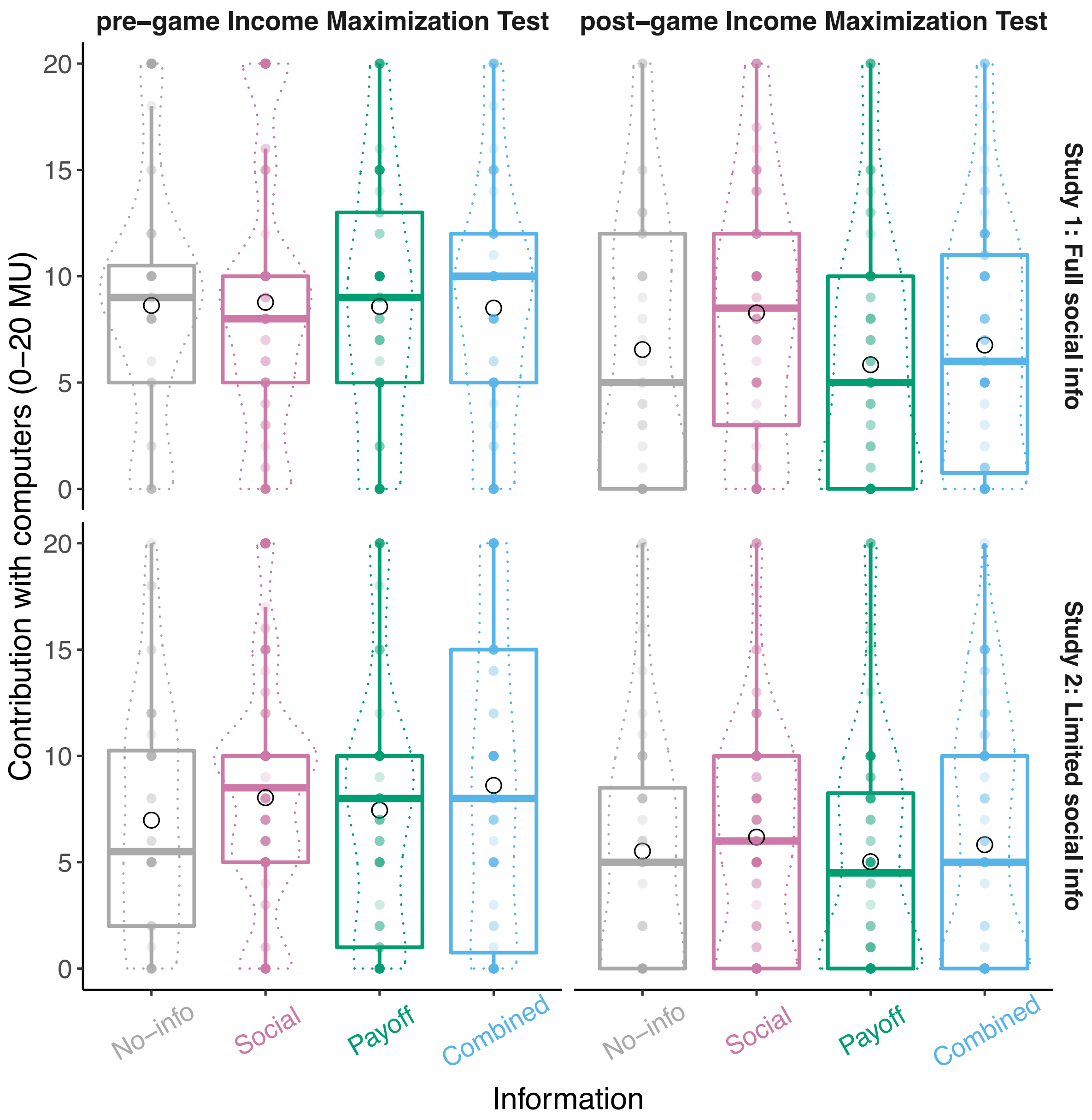

Supplementary Figure 6. Income Maximization Tests by information treatment and study. *Violin plots show distribution of contributions. Box plots show the median contribution (horizontal bar) and interquartile range. The mean contributions are shown by the empty black circles. We tested all individuals twice, one before and once after the repeated public goods game. Number of individuals (study1/study 2): No-info = 84 (40/44); Social = 212 (80/132); Payoff =160 (80/80); Combined = 160 (80/80).*

Supplementary Table 1. Linear mixed models using maximum likelihood on mean group contributions depending on information treatment and year of data collection (study 1 versus study 2).

Term \ Model[1]	M1<br>*p*-value	M2<br>*p*-value	M3[2]<br>*p*-value	M4<br>*p*-value
Game round	<0.001	<0.001	<0.001	<0.001
Treatment	0.896	0.278	0.340	0.333
Year of study	/	/	<0.001	0.005
Game round * Treatment	/	<0.001	<0.001	<0.001
Game round * Year of study	/	/	/	0.572
Treatment * Year of study	/	/	/	0.852
Game round * Treatment * Year	/	/	/	0.575
Number of observations	1,386	1,386	1,386	1,386
Number of independent units	154	154	154	154
Number of parameters	9	12	13	20
AIC	11,152	11,139	11,130	11,141
BIC	11,200	11,202	11,198	11,245
Significance of more complicated model[3]	NA	<0.001	<0.001	0.877

[1] Models controls for group identity with random effect intercepts and slopes.
[2] This is the optimal model.
[3] Model comparisons performed with a likelihood ratio test.

Supplementary Table 2. Robustness check. Results from a generalized linear mixed model fit for qualitative comparison with the results of the linear mixed model in Table 1 (reference treatment: Social).

**Fixed effects**	**Coefficient**	**S.E.**	**z**	**p-value**	**Sig.**
(Intercept) [Social]	0.12	0.145	0.8	0.428	
Game round [Social]	-0.11	0.021	-5.4	<0.001	***
Year of study [2019]	-0.41	0.138	-3.0	0.003	***
Treatment [No-info]	-0.21	0.221	-1.0	0.335	
Treatment [Payoff]	0.12	0.180	0.7	0.512	
Treatment [Combined]	0.24	0.180	1.3	0.185	
Game round*Treatment [No-info]	0.05	0.039	1.4	0.175	
Game round*Treatment [Payoff]	-0.1	0.032	-3.2	0.002	***
Game round*Treatment [Combined]	-0.1	0.032	-2.6	0.010	***
Random effects	Variance	Std. Dev.			
Group ID (intercept)	0.71	0.841			
Group ID (game round)	0.02	0.146			

Number of observations = 1,386

Number of independent groups = 154

457

Supplementary Table 3: Results of Study 1 with full social information.

**Fixed effects**	Estimate*	Std. Error	df**	t value	P value***
Reference intercept (No-info)	50.07	6.28	70.0	7.976	<.001
Social intercept	+0.32	7.69	70.0	0.042	0.967
Payoff intercept	+4.21	7.69	70.0	0.548	0.586
Combined intercept	+4.59	7.69	70.0	0.597	0.552
Reference slope (No-info)	-1.30	0.92	70.0	-1.415	0.162
Social slope	-0.04	1.12	70.0	-0.032	0.974
Payoff slope	-2.43	1.12	70.0	-2.162	0.034
Combined slope	-1.74	1.12	70.0	-1.550	0.126
Number of independent groups	70				
Number of observations	630				
Random effects					
Intercept (by group) variance	375.4	19.375			
Slope (by group) variance	6.4	2.538			
Residual	118.6	10.889			

*Response variable is the group total contribution (0-80) converted to a natural number percentage (group contribution / 0.8)

**Degrees of Freedom estimated with the Satterthwaite method

***P value is for the comparison with the reference intercept/slope

Supplementary Table 4: Results of Study 2 with limited social information.

**Fixed effect**	Estimate*	Std. Error	df**	t value	P value***
Reference intercept (No-info)	36.04	5.40	84.02	6.675	<.001
Social intercept	+7.25	6.23	84.02	1.163	0.248
Payoff intercept	+8.39	6.72	84.02	1.248	0.216
Combined intercept	+12.90	6.72	84.02	1.919	0.058
Reference slope (No-info)	-0.86	0.67	84.00	-1.283	0.203
Social slope	-1.43	0.78	84.00	-1.844	0.069
Payoff slope	-2.60	0.84	84.00	-3.113	0.003
Combined slope	-.2.91	0.84	84.00	-3.484	<.001
Number of independent groups	84				
Number of observations	756				
Random effects					
Intercept (by group) variance	316.5	17.789			
Slope (by group) variance	3.4	1.831			
Residual	96.6	9.829			

*Response variable is the group total contribution (0-80) converted to a natural number percentage (group contribution / 0.8)

**Degrees of Freedom estimated with the Satterthwaite method

***P value is for the comparison with the reference intercept/slope

Supplementary Table 5: Generalized linear model analysing the second Income Maximization Test played with computers (IMT2).

Fixed effect^	Estimate [95% CI]*	df	t value	P value
Reference intercept (treatments with no payoff information)	-1.5 [-1.78, -1.29]	612	-12.327	<0.001
Treatments with payoff information	-0.27 [-0.47, -0.07]	612	-2.621	0.009
Study 2 intercept	-0.24 [-0.45, -0.04	612	-2.361	0.019
IMT1	0.11 [0.10, 0.13]	612	12.734	<0.001
Number of individuals (observations)	616 (1,232)			

^Dispersion parameter for quasibinomial family taken to be 6.277245.

^Null deviance: 5788.2 on 615 degrees of freedom.

^Residual deviance: 4582.0 on 612 degrees of freedom.

*Raw coefficients, non-exponentiated.
